# Berardinelli-Seip syndrome and achalasia: a shared pathomechanism?

**DOI:** 10.1007/s00431-015-2556-y

**Published:** 2015-05-22

**Authors:** Rachel J. van der Pol, Marc A. Benninga, Jocelyne Magré, Lionel Van Maldergem, Joost Rotteveel, Marjo S. van der Knaap, Tim G. de Meij

**Affiliations:** 1 Department of Pediatric Gastroenterology and Nutrition, Emma Children’s Hospital AMC, Amsterdam, The Netherlands; 2 INSERM UMR_S938, Université Pierre et Marie Curie, Paris, France; 3 INSERM UMR_S1087, L’Institut du Thorax, Nantes, France; 4 Centre de Génétique Humaine, Université de Franche-Comté, Besançon, France; 5 Department of Pediatric Endocrinology, VU University Medical Center, Amsterdam, The Netherlands; 6 Department of Pediatric Neurology, VU University Medical Center, Amsterdam, The Netherlands; 7 Department of Pediatric Gastroenterology, VU University Medical Center, Amsterdam, The Netherlands

**Keywords:** Berardinelli-Seip congenital lipodystrophy, Achalasia, Manometry

## Abstract

Berardinelli-Seip congenital lipodystrophy (BSCL) is an uncommon autosomal recessive disorder. Patients with BSCL present with a distinct phenotype since subcutaneous fat is largely lacking and musculature has become more prominent. During childhood, diabetes and acanthosis nigricans evolve and female patients may develop hirsutism. Different genes encoding this entity have been described. Achalasia is a rare esophageal motility disorder, characterized by its distinct motility pattern with absent or incomplete lower esophageal sphincter (LES) relaxations. The exact cause of achalasia is yet unknown. Here, we describe a patient with achalasia in the context of BSCL, which might be linked by a shared pathophysiologic background, as evaluated in this case report.

*Conclusion*: In a BSCL patient presenting with gastrointestinal symptoms, a motility disorder of the gastrointestinal tract should be considered.

**Table:** 

**What is Known:** • *Berardinelli-Seip congenital lipodystrophy (BSCL) and achalasia are both disorders characterized by low prevalence.*
**What is New:** • *Co-existence of both diseases is described in this report. Linkage by a potential common pathophysiologic background is discussed in this paper.*

**What is Known:**

• *Berardinelli-Seip congenital lipodystrophy (BSCL) and achalasia are both disorders characterized by low prevalence.*

**What is New:**

• *Co-existence of both diseases is described in this report. Linkage by a potential common pathophysiologic background is discussed in this paper.*

## Introduction

Berardinelli-Seip congenital lipodystrophy (BSCL) is an autosomal recessive disorder, first described by Berardinelli in 1954 and subsequently by Seip in 1959. The prevalence is estimated to be less than 1 per 12 million people [6]. Patients with BSCL usually present with a distinct phenotype, characterized by lack of subcutaneous fat and presence of prominent musculature. Besides, infants may present with an increased appetite, hepatosplenomegaly, and umbilical hernia. During childhood, diabetes as well as acanthosis nigricans evolves, and in female patients, hirsutism may develop [7]. Mutations in *AGPAT2* (*OMIM#603100*) on chromosomes 9q34 [1] or *BSCL2* (*OMIM#606158*) on chromosome 11q12.3 [10] account for 95 % of BSCL cases [11]. *AGPAT2* encodes the enzyme acyltransferase 1-acylglycerol-3-phosphate-O-acyltransferase 2, involved in the cascade of the biosynthesis of triacylglycerides and glycerophospholipids [1]. *BSCL2* encodes the protein seipin. Mutations in this gene disrupt the normal development and function of adipocytes and prevent normal storage of fat in lipid droplets [3]. In a minority of cases, biallelic mutations in one or two genes encoding major components of the caveolae, caveolin 1 (*CAV1, OMIM#601047*) [9], polymerase I and transcript release factor (*PTRF OMIM#613327)*, are present. Mutations in a gene encoding an important transcription factor of adipocyte differentiation, the *peroxisome proliferator-activated receptor gamma* (PPARG) (*OMIM#613327)*, may also cause BSCL [4].

Achalasia is a rare esophageal motility disorder, characterized by its distinct motility pattern with absent or incomplete lower esophageal sphincter (LES) relaxation. Typical presenting symptoms include dysphagia for solids and eventually liquids, regurgitation, heartburn, and weight loss. Diagnosis of achalasia is confirmed by performing high-resolution manometry (HRM) showing the absent or incomplete LES relaxations and is further classified in subtypes by different aberrant motility patterns of the esophageal body [2]. Barium swallow studies can be performed to show dilatation of the esophagus or, typical for achalasia, a bird beak appearance of the distal esophagus. The exact cause of achalasia remains to be elucidated yet, but decreased or absent myenteric neurons of the distal esophagus play a key role in the pathogenesis. Influence of genetic and immunologic factors has also been suggested to be involved [14]. Twin concordance seems significant, but the nature of the genetic contribution is still inconclusive. Additionally, achalasia may present as part of genetic syndromes or may occur in association with other abnormalities or diseases, such as Down syndrome and Allgrove syndrome. In this case report, we describe a patient with achalasia in the context of BSCL.

## Case report

**Fig. 1:**
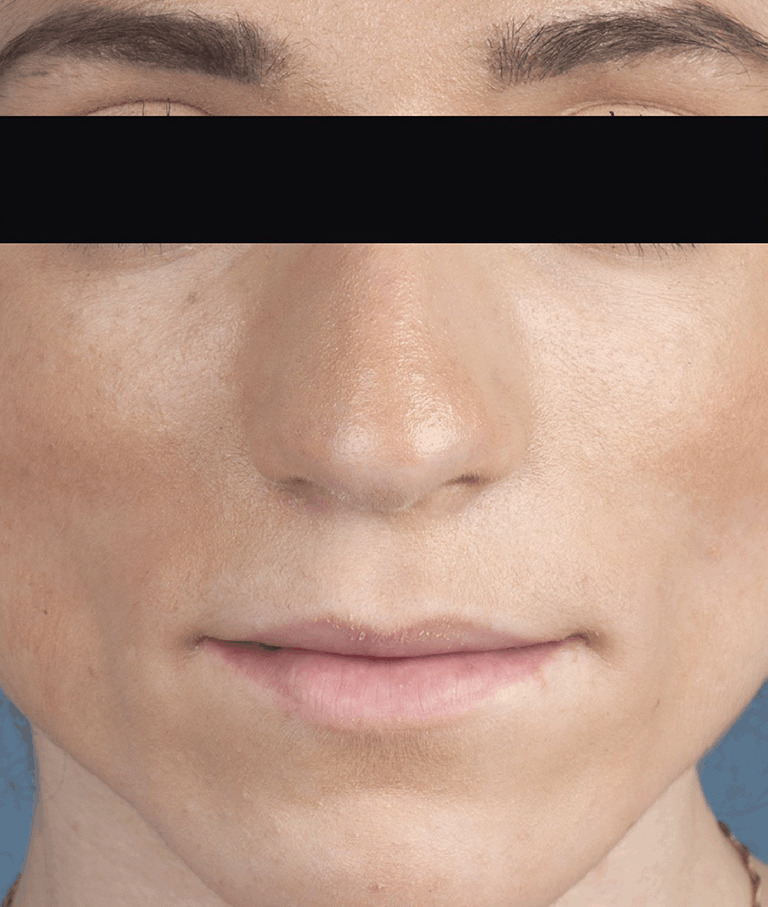
Clinical picture of presented patient, demonstrating reduced subcutaneous fat tissue

A 14-year-old Turkish girl presented with progressive dysphagia, odynophagia, regurgitation, nausea, and vomiting. Her history revealed congenital hip dysplasia. From the age of 2 months, she had failure to thrive, despite increased appetite. When she was 6 years old, she was clinically diagnosed with BSCL, because of generalized muscular hypertrophy, macroglossia, lack of subcutaneous fat, hirsutism, percussion-induced myoedema (localized transient swelling of muscle induced by percussion without electrical activity at electromyography, excluding myotonia), and hepatosplenomegaly. Patient was third child of not knowingly consanguineous parents, but they originated from two close villages. A picture of the patient, currently 18 years old, is displayed in Fig. 1. At that time, laboratory analysis showed elevated serum creatine kinase levels (2305 U/L), hypertriglyceridemia (4.6 mmol/l), and hyperinsulinism (330 pmol/l) with normoglycemia, indicating insulin resistance. Ultrasound of the abdomen confirmed hepatosplenomegaly with signs of hepatic steatosis and revealed no other abnormalities. Because of the diagnosis BSCL, a low fat with medium-chain triglycerides and slow-release carbohydrates diet was prescribed. Genetic analysis revealed no pathogenic mutations in *AGPAT2*. At the age of 12 years, the diagnosis of BSCL was reconsidered. No mutations in *BSCL2*, *AGPAT2*, or *CAV1* were detected and subsequently molecular screening of *PTRF* was performed. A homozygous c.258C>T transition was detected in *PTRF* exon 1, predicting a p.Q87X nonsense mutation. This mutation was found in heterozygous state in both parents and her 15-year-old asymptomatic brother. This mutation has not yet been reported in the ExAC database of 60,000 exomes and was absent in 100 unrelated control subjects.

**Fig. 2:**
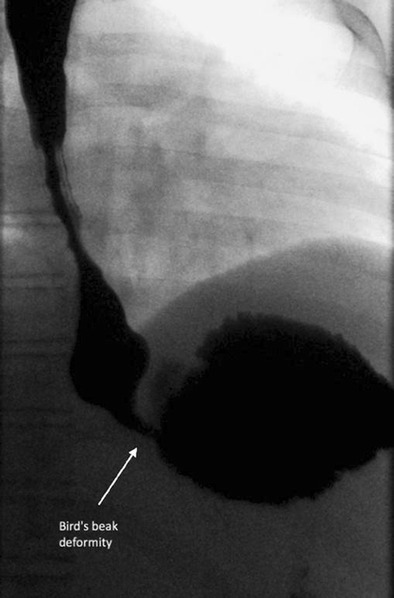
Upper esophageal series with barium, showing a dilated esophagus body with stasis of the contrast and “bird’s beaking” (*arrow*), suggestive of achalasia

**Fig. 3:**
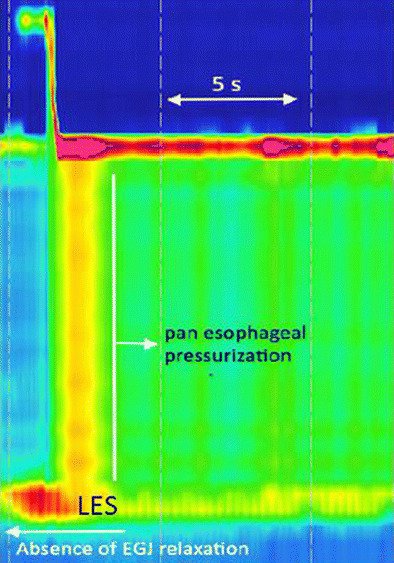
High-resolution manometry, showing a pressure topography plot during a swallow. Horizontal axis corresponds with time, vertical axis with length along the esophagus. Pressure magnitude is encoded in *colors*; lower pressure regions are denoted by *blue color*, with increasing pressure indicated by *green to yellow colors*. High-pressure regions are denoted by *red color. Continuous yellow color* along the esophagus illustrates pan-esophageal pressurization without esophageal peristaltic contractions. Absence of LES relaxations is illustrated by a continuous high pressure (*horizontal yellow line*) without a “break,” e.g., relaxation, which is characteristic for achalasia. *EGJ* esophagogastric junction, *LES* lower esophageal sphincter, *s* seconds)

At time of presentation, she complained of progressive dysphagia and vomiting. Upper esophageal series with barium were performed under suspicion of achalasia. A dilated esophageal body, with stasis of the contrast in the esophagus, was seen. At the esophagogastric junction, a bird’s beak deformity was observed (Fig. 2). HRM (Manoscan360™, Given® imaging) showed absent and incomplete LES relaxations, with an integrated relaxation pressure (IRP) of 55.6 mmHg, above the 15-mmHg threshold according to the Chicago classification for achalasia in adults [2]. Peristaltic contractions of the esophagus were absent or simultaneous (in 20 % of swallows). Moreover, pan-esophageal pressurization and spastic contractions were seen (Fig. 3). Based on these typical findings on HRM, the diagnosis achalasia was confirmed. Hence, pneumodilatation of the LES (with a 30-mm balloon) was performed with immediate relief of the dysphagia symptoms. During later follow-up, complaints of dysphagia returned and nocturnal coughing became bothersome. Repeated pneumodilatations of the LES were eventually effective, and dysphagia completely resolved. Recently, complaints of dysphagia started to return gradually. Meanwhile, she developed episodes of hyperglycemia with increased HbA1c levels (up to 9.3 %) despite carbohydrate restricted diet, caused by insulin resistance, for which metformin and Levemir were prescribed.

## Discussion

Since esophageal dilatation and weakness has previously been observed in patients with *PTRF* gene mutation, it could be hypothesized that BSCL is associated with abnormalities of esophageal motility, like achalasia. MEDLINE was searched using the terms congenital lipodystrophy, Berardinelli-Seip, and achalasia (Mesh and all fields). The co-existence of BSCL and achalasia has not yet been described in the current literature.

**Fig. 4:**
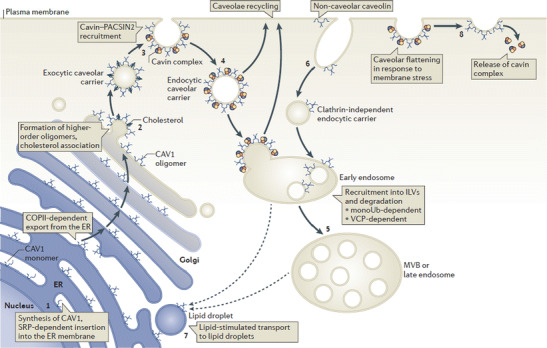
Membrane trafficking of caveolins through exocytosis and endocytosis. Schematic showing the itinerary of caveolin 1 (*CAV1*) from synthesis in the rough endoplasmic reticulum (*ER*) (*1*) and its trafficking through the Golgi complex (*2*) to the cell surface. The cavin–PKC and casein kinase substrate in neurons 2 (*PACSIN2*) complex has a vital role in stabilizing caveolae at the plasma membrane (*3*). Upon endocytosis (*4*), caveolae can be targeted to early endosomes and can then be recycled back to the plasma membrane or targeted to multivesicular bodies (*MVBs*) for degradation (*5*). Targeting to MVBs requires monoubiquitylation (*monoUb*) of caveolin and is valosin-containing protein (*VCP*)-dependent. Non-caveolar caveolin can be internalized through other endocytic pathways (*6*). Caveolin can also traffic to lipid droplets, although the route by which this is mediated is unclear (*dashed lines*) (*7*). Cavins may be released from caveolae into the cytoplasm upon flattening of caveolae (*8*). *COPII* coat protein II, *ILV* intralumenal vesicle, *SRP* signal recognition particle [12]. (printed with permission from publisher)

In our patient, a homozygous mutation in *PTRF* caused BSCL. Clinical features of patients with *PTRF* mutation include generalized lipodystrophy, distal myopathy, muscular hypertrophy, percussion-induced muscle mounding (myoedema), elevated serum creatine kinase concentration, cardiac arrhythmias, hypertriglyceridemia, insulin resistance, and normal intelligence. All these clinical findings, with the exception of cardiac abnormalities, were present in our patient. PTRF plays a pivotal role in the formation of caveolae, bulb-shaped invaginations of the plasma membrane. These structures contribute to clathrin-independent endocytosis, signal transduction, as well as cholesterol transport and regulation. In Fig. 4, the membrane trafficking process of caveolins through exocytosis and endocytosis is displayed [12]. Three types of caveolins subtypes have been identified so far: caveolin-1, 2, and 3. Caveolin-1 is involved in the process of binding fatty acids and their translocation into lipid droplets [10]. Types 1 and 2 are expressed together in many cells, including adipocytes, endothelial, and smooth muscle cells, and type 3 in striatal (skeletal and cardiac) muscle cells [8]. Caveolin-1 dysfunction appears to be at cause in the alteration of lipids metabolism determining an abnormal profile of plasma lipids and lipoatrophy observed in BSCL patients [9]. Sarcolemmal caveolin-3 impairment secondary to the *PTRF* mutation is possibly at cause of the distinctive muscle phenotype observed in this subtype of BSCL.

Serum creatine kinase elevation appears to be an interesting laboratory marker for diagnosis of BSCL due to *PTRF* mutations [8].

So far, 21 patients with *PTRF* mutations have been described in the literature. In two of these patients, esophageal dysfunction has been described: esophageal dilatation [8] in one case and esophageal weakness in the other [13]. However, details of the diagnostic work-up were not specified in the corresponding reports. Different hypotheses can be proposed to explain esophageal dysfunction in the context of BSCL. First, dysfunction of caveolin-1 might lead to alterations of the interstitial cells of Cajal (ICC), leading to impaired function of nitric oxide (NO) [5, 8]. In rats, it has been shown that relaxation of the LES is mediated by NO. Since achalasia patients have injured inhibitory nerves and ICC are suggested to form functional units with nitrergic nerves, ICC have been studied as potential link in the pathophysiology of achalasia [15]. However, damage of ICC in achalasia was not related to injury of nitrergic nerves and duration of disease [16]. Perhaps, a more likely explanation is found in the sustained, electrically silent muscle contractions as part of BSCL, found also in this patient. This would be a novel pathophysiological mechanism, related to BSCL, which obviously needs to be confirmed in future studies.

In conclusion, this case report describes a patient presenting with two rare disorders, which might be linked by a common pathophysiologic background. However, insufficient data are currently available to explain how these two disorders are exactly related. Other pathways, yet to be clarified, should be investigated in further studies. In a BSCL patient presenting with gastrointestinal symptoms, a motility disorder of the gastrointestinal tract should be considered.

## Abbreviations

BSCL: Berardinelli-Seip congenital lipodystrophy
CAV1: Caveolin 1
HRM: High-resolution manometry
ICC: Interstitial cells of Cajal
LES: Lower esophageal sphincter
PTRF: Polymerase I and transcript release factor
